# Pharmacological modulation of Kv3.1 mitigates auditory midbrain temporal processing deficits following auditory nerve damage

**DOI:** 10.1038/s41598-017-17406-x

**Published:** 2017-12-13

**Authors:** Anna R. Chambers, Nadia Pilati, Pooja Balaram, Charles H. Large, Leonard K. Kaczmarek, Daniel B. Polley

**Affiliations:** 1 Eaton-Peabody Laboratories, Massachusetts Eye and Ear Infirmary, Boston, MA USA; 2 Autifony SRL, Verona, Italy; and Autifony Therapeutics Limited, Imperial College Incubator, London, UK; 3 Department of Otolaryngology, Harvard Medical School, Boston, MA USA; 4 Departments of Pharmacology and Cellular and Molecular Physiology, Yale University School of Medicine, New Haven, CT USA; 5 Present Address: Institute of Basic Medical Sciences, University of Oslo, Oslo, Norway

## Abstract

Higher stages of central auditory processing compensate for a loss of cochlear nerve synapses by increasing the gain on remaining afferent inputs, thereby restoring firing rate codes for rudimentary sound features. The benefits of this compensatory plasticity are limited, as the recovery of precise temporal coding is comparatively modest. We reasoned that persistent temporal coding deficits could be ameliorated through modulation of voltage-gated potassium (Kv) channels that regulate temporal firing patterns. Here, we characterize AUT00063, a pharmacological compound that modulates Kv3.1, a high-threshold channel expressed in fast-spiking neurons throughout the central auditory pathway. Patch clamp recordings from auditory brainstem neurons and *in silico* modeling revealed that application of AUT00063 reduced action potential timing variability and improved temporal coding precision. Systemic injections of AUT00063 *in vivo* improved auditory synchronization and supported more accurate decoding of temporal sound features in the inferior colliculus and auditory cortex in adult mice with a near-complete loss of auditory nerve afferent synapses in the contralateral ear. These findings suggest modulating Kv3.1 in central neurons could be a promising therapeutic approach to mitigate temporal processing deficits that commonly accompany aging, tinnitus, ototoxic drug exposure or noise damage.

## Introduction

Cochlear frequency processing can be conceptualized as a limited-resolution ‘filter bank’ that decomposes broadband sounds into a spatially organized array of narrowband signals. Multiplexed temporal fine structure and envelope cues from single cochlear filters are encoded by individual Type-I spiral ganglion neurons (SGNs) with firing rates that can exceed 1 kHz. Extracting the essential features from each auditory nerve’s afferent barrage requires neural circuit elements in the auditory brainstem that can encode sub-millisecond timing differences with high fidelity. Auditory brainstem and midbrain neurons meet these demands by expressing unique synaptic architecture, precisely tuned inhibitory circuits and a variety of biophysical specializations such as the expression of ion channels with rapid activation kinetics^1–6^.

Aging, ototoxic drugs or even moderate levels of environmental noise exposure can eliminate Type-I SGN synapses onto cochlear hair cells, thereby reducing the bandwidth of information that can be transmitted from the ear to the brain^7–10^. At the level of the auditory cortex (ACtx), neural circuits compensate for cochlear afferent loss by decreasing local inhibitory tone and increasing the central gain on diminished afferent signals^11–15^. This compensatory plasticity restores higher auditory coding and perceptual awareness of basic auditory features that can be encoded by variations in overall firing rate, but offers comparatively little benefit for the fine-grained temporal analysis that is uniquely performed by specialized auditory brainstem and midbrain circuits^11,12^. The principal motivation for this study is to ask whether temporal processing deficits arising from the selective loss of auditory nerve afferent fibers can be ameliorated with drugs that modulate the biophysical properties of fast-spiking neurons in subcortical auditory nuclei.

The amount and type of voltage-gated potassium (Kv) channels expressed in the cell membrane are major determinants of its intrinsic electrical excitability. Kv channels control the resting membrane potential as well as the shape, number, rate and timing of action potentials initiated in response to a stimulus. Kv3.1, a member of the *Shaw* class of Kv channels, is a high-threshold delayed rectifier channel that is widely expressed in fast spiking neurons throughout the auditory brainstem^16–18^. Kv3.1 rapidly repolarizes the membrane potential during an action potential, effectively shortening the refractory period and thus enabling neurons to sustain high firing rates in response to high-frequency synaptic inputs^19–21^. Pharmacological, computational or genetic elimination of Kv3.1 currents broadens the spike width and renders neurons unable to follow rapid trains of direct current injections^22–25^. Interestingly, the effect of down-regulating Kv3.1 currents is more than a theoretical curiosity, as the protein levels, phosphorylation state and channel conductances of Kv3.1 are all regulated by auditory afferent input in auditory brainstem nuclei^26–30^. Here, we focus on the effects of increasing Kv3.1 currents with AUT00063, a recently developed compound that enhances Kv3.1 conductance by shifting the voltage-dependence of activation of the channels to more negative potentials^31^.

The goals of this study are to characterize auditory temporal coding deficits in the inferior colliculus (IC), an auditory midbrain nucleus, following a selective, near-complete elimination of auditory nerve afferent fibers in adult mice. We present evidence that the Kv3.1 modulator, AUT00063, selectively improves spike precision and reliability in fusiform neurons of the dorsal cochlear nucleus that provide a principal input to the inferior colliculus. Using a simple *in silico* simulation, we demonstrate that computational enhancement of Kv3.1 currents can improve the temporal synchronization accuracy of model neurons. After determining that the central auditory pathway of denervated mice express high levels of Kv3.1, we return to the intact preparation and ask whether systemic administration of AUT00063 can rapidly improve temporal coding fidelity in the IC of awake mice as well as downstream auditory areas.

## Results

### Ouabain treatment leads to selective degeneration of Type-I spiral ganglion neurons

**Figure 1:**
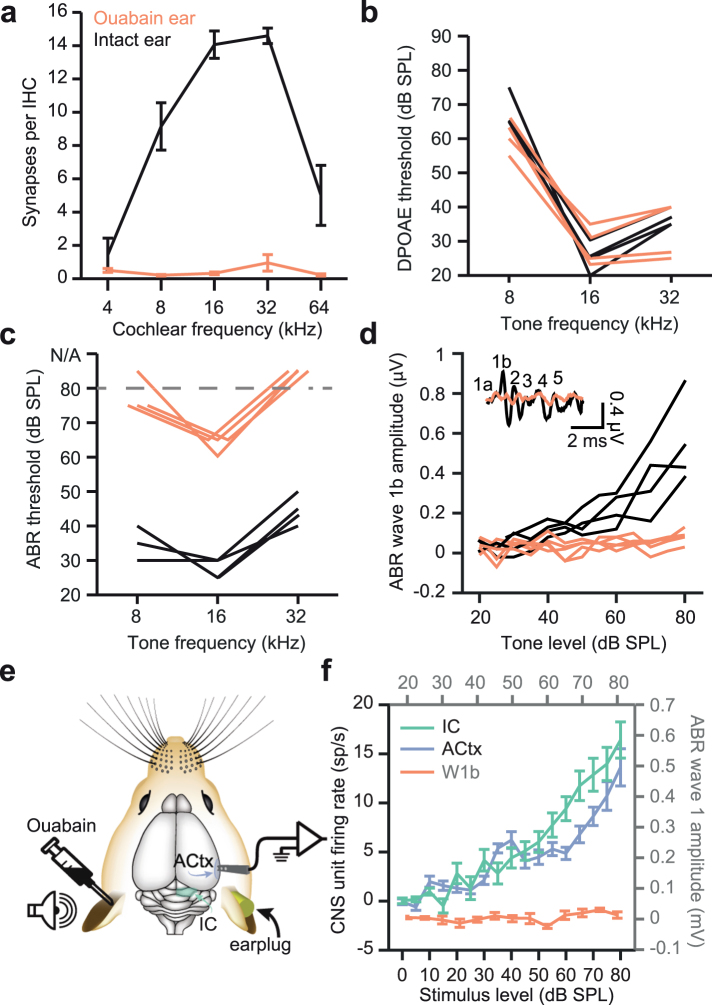
Ouabain treatment selectively degenerates spiral ganglion neurons, eliminating the ABR without affecting cochlear amplification. (**a**) Cochlear afferent synapses (operationally defined from juxtapositions of inner hair cell synaptic ribbons and auditory nerve glutamate receptors) were made from immunohistochemically stained sections corresponding to cochlear frequency locations. In ouabain treated ears (n = 4 mice, red line) compared to control (black line), more than 95% of synapses were eliminated by ouabain treatment at least 30 days prior. (**b**) Hair cell function was preserved 30 days after ouabain treatment, as shown via DPOAE threshold measurements taken at various F2 frequencies. Thresholds from the ouabain-treated left ear (red) and untreated right ear (black) are shown from individual mice after ouabain treatment (n = 4 ears). (**c**) Auditory brainstem response (ABR) wave 1b threshold measurements were significantly higher from the ouabain-treated ear, indicating a near-complete loss of auditory nerve fibers. (**d**) ABR wave 1b (inset) amplitudes show a barely detectable or absent signal from the ouabain-treated ear. (**e**) Schematic of midbrain and cortex recording experiments. Adult mice treated with ouabain 30 days prior were implanted with chronic recording electrodes (see Methods) in the IC and ACtx contralateral to the ouabain-treated (left) ear. (**f**) Although ABR measurements indicated a near-complete loss of activity in the auditory nerve, central stations of the auditory pathway showed robust responsiveness to sound and monotonically increasing rate-level functions (green line = IC; blue line = ACtx). All data in Fig. 1 have been published in an earlier report^12^.

**Figure 2:**
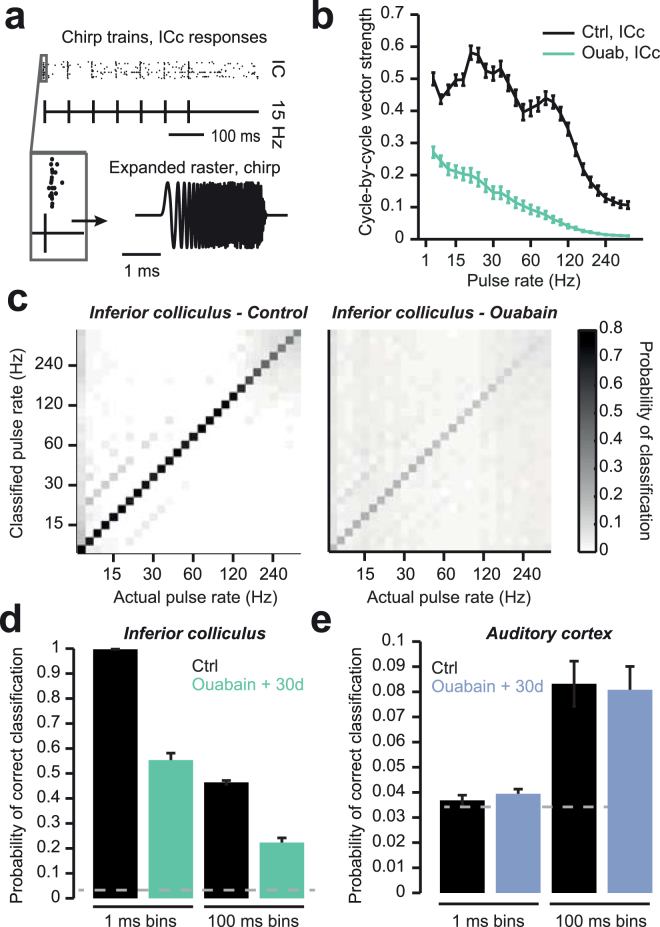
Auditory nerve damage is associated with profound impairments in auditory temporal processing. (**a**) Schematic of chirp train stimulus and example raster of IC responses to a 15 Hz pulse train. (**b**) Vector strength measures the synchronization of the neural response to a temporally modulated stimulus. Synchronization is reduced in the ICc 30 days after ouabain (green line) compared to sham-treated control mice (black line). (**c**,**d**) A PSTH-based minimal Euclidean distance classifier (see Methods) was used to classify chirp train frequencies according to multiunit responses. Average confusion matrices (bin size = 10 ms) for control- (left) and ouabain-treated mice (right) show a significant decrease in chirp train classification performance after ouabain that did not recover to control levels with 30 days of recovery in the ICc. Average correct classification in the ICc (**c**) and ACtx (**d**) in control- and ouabain-treated mice when using PSTH bin sizes of 1 or 100 ms. Ensembles of 20 units were utilized for the classification analysis. All data in Fig. 2 have been published in an earlier report^12^.

Severe cochlear neuropathy was induced in adult CBA/CaJ mice with unilateral round window applications of ouabain, a Na^+^/K^+^ ATPase pump inhibitor that has been shown to selectively eliminate Type-I SGNs without damaging sensory and non-sensory cells in the cochlea^32,33^. The data presented in Figs 1 and 2 are a subset of data that have been reported in an earlier study on these mice, but are reanalysed and included here to put the characteristics of auditory nerve damage and baseline compensatory recovery prior to drug treatment into context^12^. Histological analysis and cochlear function testing reveal that application of ouabain to the cochlear round window eliminated over 95% of Type I spiral ganglion afferent synapses (as assessed by synaptic counts between hair cells and Type I SGNs, n = 349 inner hair cells in ouabain-treated and 372 inner hair cells in control ears, F(1) = 345.7, *p* < 0.01, repeated-measures ANOVA, Fig. 1a) without damaging cochlear hair cells, as inferred from normal distortion product otoacoustic emission (DPOAE) thresholds between the treated and untreated ear (F(1) = 1.36, p = 0.27, repeated-measures ANOVA, Fig. 1b). The loss of Type-I SGNs was reflected in the substantially higher tone-evoked auditory brainstem response (ABR) threshold (F(1) = 371.1, p < 0.01, repeated-measures ANOVA, Fig. 1c) and the virtual elimination of ABR wave 1b (Fig. 1d, F(1) = 13, p < 0.01, repeated-measures ANOVA).

### IC and ACtx units recover low thresholds and steep rate-level functions after one month of recovery, however temporal response properties are persistently degraded

Previous studies have shown a progressive recovery of forebrain sound processing and auditory detection behavior despite the elimination of >95% of cochlear afferent synapses and the virtual absence of acoustic reflexes and auditory brainstem response^11–13^. To contrast the recovery of spike rate-based and spike timing-based coding of sound features after auditory nerve damage, we implanted extracellular recording probes into the central nucleus of the inferior colliculus (ICc) and ACtx contralateral to the ouabain-treated (or for control recordings, sterile water-treated) ear (Fig. 1e). Recordings were then performed in awake, passively listening mice while sounds were presented to the ouabain-treated ear. To restrict acoustic stimulation to only one (control vs. ouabain-treated) ear, we fitted a dense foam earplug into the ipsilateral, intact ear. In previous experiments, we confirmed that the combination of the earplug and head shadow provides at least 60 dB of attenuation, thereby ensuring that sound-evoked responses reported here is exclusively mediated by the small fraction of surviving nerve fibers in the contralateral, ouabain-treated ear^12,34^.

Input/output response functions were derived from presenting broadband chirp stimuli across a range of sound levels and either measuring the amplitude of ABR wave 1b or the spiking rate from multiunit clusters recorded simultaneously from ICc (n = 80 recording sites) and ACtx (n = 76 recording sites)^35^. Averaged rate-level functions from multiunit recordings in the IC and ACtx showed that 30 days after contralateral Type-I SGNs were nearly eliminated with ouabain, the sound-evoked growth function from the auditory nerve is essentially flat, while growth functions from higher stages of central processing exhibit relatively normal response thresholds and steeply increasing response slopes^11–13^ (Fig. 1f). However, when the presentation rate of broadband chirps was varied, rather than the sound level, pronounced central processing deficits were apparent (Fig. 2). For these experiments, chirp trains (1–320 Hz, 500 ms in duration) were presented 20 dB above unit response threshold, to account for any differences in sensation level between control and ouabain-treated recordings (Fig. 2a). Cycle-by-cycle vector strength, a metric that takes into account both the degree of synchronization and likelihood of sustained synchronization through the duration of a temporally modulated stimulus^36^, was calculated from the multiunit responses to chirp trains. Compared to animals treated with round window applications of sterile water, ICc spike trains recorded from ouabain-treated mice exhibited significantly lower synchronization despite robust sound-evoked onset responses (black versus green line, Fig. 2a,b, F(1) = 77.9, *p* < 0.01, repeated-measures ANOVA).

To estimate how the loss of synchronization might impact the encoding of stimulus chirp rate, we implemented a PSTH-based decoder that attempted to classify chirp rate based on the patterns of multiunit spiking across small ensembles of recording sites (n = 20 units per ensemble)^37^. In recordings from sham-treated mice, the neural classifier accuracy was almost at ceiling, correctly identifying the stimulus pulse rate, with only a small number of errors at the highest rates (Fig. 2c, left). In recordings from ouabain-treated mice, classification accuracy was greatly impaired across the full range of pulse rates (Fig. 2c, right).

To estimate the importance of spike timing on stimulus decoding, the mean classification accuracy was determined by binning the PSTH at fine or coarse resolution (1 versus 100 ms bins, respectively). In the ICc, chirp rate classification was significantly impaired using both bin sizes (*p* < 0.01, unpaired t-tests for both bin sizes; Fig. 2d). In ACtx, we did not observe a significant difference in classification performance with either bin size (Fig. 2e, *p* = 0.15 (1 ms bins), *p* = 0.6 (100 ms bins), unpaired t-tests)). These results suggest that the recovery of temporal processing in ACtx may be more complete than in ICc, as has previously been described for the coding of sound frequency and sound level^11,12^. On the other hand, far less recovery is needed to restore normative temporal processing in ACtx because temporal decoding accuracy is poor to begin with.

### AUT00063, a positive modulator of the Kv3.1 delayed rectifier channel, improves temporal precision of auditory neuron spiking *in vitro*

As a next step, we asked whether it might be possible to enhance temporal processing deficits through pharmacological modulation of the intrinsic electrical properties of subcortical auditory neurons. We focused on pharmacological modulation of Kv3.1, both because it has a well-established role in supporting sustained, high-fidelity spike trains firing at high rates and also because Kv3.1 expression levels in the auditory brainstem are strongly modulated by sound deprivation or enrichment^17,26,27,29^. We characterized the effects of AUT00063, which shifts the voltage-dependence for activation of Kv3.1 to more negative potentials and slows the rate of deactivation of Kv3.1 currents, similar to previously reported compounds, AUT1 and AUT2^31,38^.

**Figure 3:**
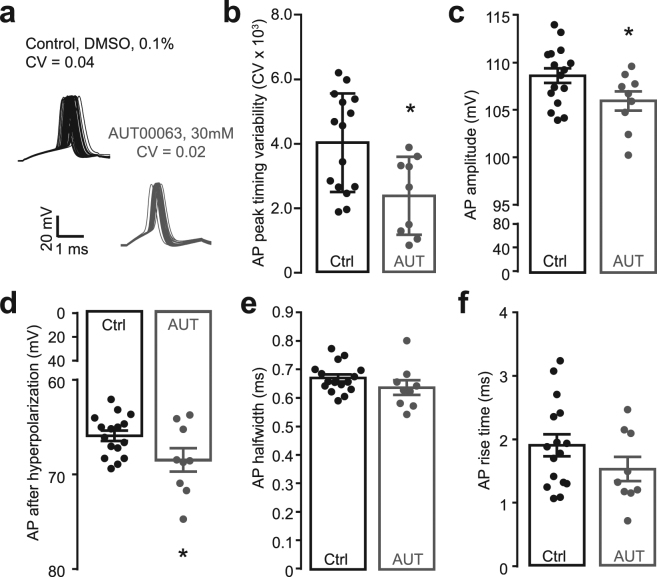
Enhancing Kv3.1 currents through bath application of AUT00063 modifies action potential characteristics and improves spike timing precision. Current clamp recordings were made from individual fusiform neurons in an acute slice preparation of the dorsal cochlear nucleus. (**a**) Examples of fusiform neuron action potential (AP) waveforms in response to a single current step (800 pA at 0.6 Hz) in the presence of a control solution (0.1% DMSO, black) or AUT00063 (30 µM, gray). (**b**) Action potential variability, measured as the coefficient of variation (CV) in peak latency is significantly reduced with AUT00063. (**c–f**) Some aspects of AP shape including peak amplitude (**c**) and peak negativity of the afterhyperpolarization (**d**) were significantly modified by AUT00063, whereas AP halfwidth (**e**) and rise time (**f**) wre not. Data in *B-F* present individual fusiform neurons as single data points alongside the mean ± s.e.m. Asterisks indicate p < 0.05 with an unpaired t-test.

We evaluated the effect of AUT00063 on action potential timing by recording from individual fusiform neurons (FNs), a primary input to ICc neurons, in an acute slice preparation of the mouse dorsal cochlear nucleus. We found that bath application of AUT00063 (30 µM) significantly reduced variability of FN action potentials in response to a single current step, as compared to the effect of applying a control solution (0.1% DMSO, unpaired t-test, p = 0.011, Fig. 3a,b). FN action potential peak amplitude was reduced by AUT00063, as would be expected from a drug that increases Kv3.1 currents at less depolarized membrane potentials (unpaired t-test, p = 0.049, Fig. 3c). Enhancing Kv3.1 currents through AUT00063 also increased the negativity of the action potential afterhyperpolarization, as expected (unpaired t-test, p = 0.039, Fig. 3d). Action potential half width and rise time, which are either not shaped or only partially shaped by Kv3.1 conductances, were not different with AUT00063 versus control (unpaired t-tests, p = 0.2 and 0.18, Fig. 3e,f, respectively). Likewise, the resting membrane potential was not different between AUT00063 and control conditions (**−**52.80 ± 1.18 mV, n = 10 and **−**52.80 ± 1.18 mV, n = 10, respectively, unpaired t-test, p = 0.23).

### Computational modeling of altered potassium currents indicates improved temporal encoding by AUT00063

**Figure 4:**
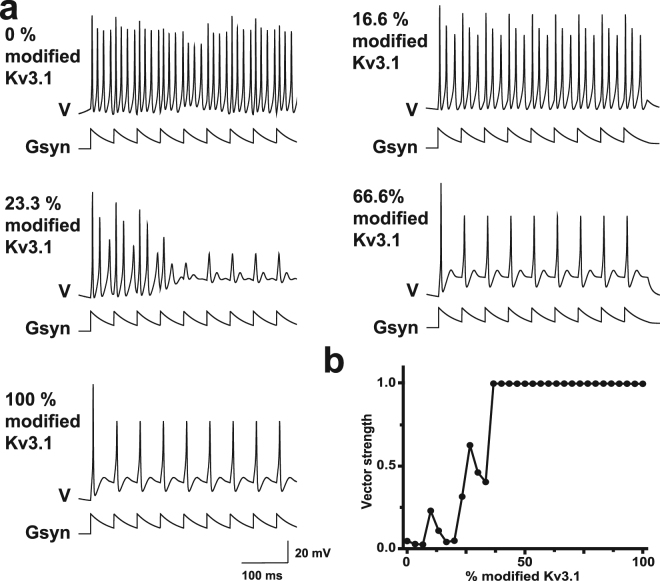
A numerical simulation suggests that increased Kv3.1 currents improves spike synchronization to periodic synaptic inputs. (**a**) A simplified computer model based on voltage-dependent Na^+^ and K^+^ currents was used to predict firing patterns (V) in response to a 20 Hz train of synaptic inputs (Gsyn). The effects of AUT00063 were simulated by computationally mixing low-threshold current from AUT-modified channels to the normal high-threshold Kv3.1 currents at levels ranging from 0–100%. (**b**) Precise synchronization to the 20 Hz synaptic input, as estimated from the vector strength statistic, improves with the addition of modified low-threshold Kv3.1 currents.

We then carried out numerical simulations to access the likely effects of AUT00063 on spike patterning to periodic synaptic inputs. We used a simplified neuronal model that is comprised of only a voltage-dependent Na^+^, current, and a K^+^ current. The characteristics of the Na+ current matched those used in previous models of auditory neurons, while the kinetic parameters for the K+ current were those used previously for Kv3.1 currents^5,22,23,28,31,39,40^. The model was stimulated with a 20 Hz train of excitatory synaptic currents, where each synaptic conductance decayed with a time constant of 40 msec. With suprathreshold stimulation, the model neurons fired repetitively throughout the stimulus train (Fig. 4a).

We progressively replaced the Kv3.1 conductance in the standard neuronal model with a modified conductance that is based on the effects of AUT compounds at comparable concentrations^31^. For simplicity, we shall term the normal Kv3.1 current “standard K+ current”, and that which was modified by AUT00063 “AUT-modified K+ current”. A low level of conversion of Kv3.1 from standard to AUT-modified reduced the number of action potentials evoked during the stimulus train (2nd trace, 16.6% modified in Fig. 4a). A higher proportion of the AUT-modified current produces adaptation of firing rate during the train (3rd trace, 23.3% modified in Fig. 4a). Above a certain threshold (~48% conversion of standard to AUT-modified), the response pattern of the neurons changes entirely, with each synaptic stimulus evoking a single action potential tightly locked to the onset of the stimulus. This transformation is reflected in the strength of the phase vector that links the onset of each synaptic stimulus to the timing of individual action potentials. With little or no modified low-threshold current, the calculated vector strength is close to zero, indicating that the timing of action potentials during the stimulus train is unrelated to the timing of individual stimuli (Fig. 4b). With higher levels of modified low-threshold current, however, each evoked action potential is faithfully locked to the onset of each synaptic input, and the phase vector is close to 1 (Fig. 4b).

### Kv3.1 mRNA in the auditory midbrain and cortex of mice

**Figure 5:**
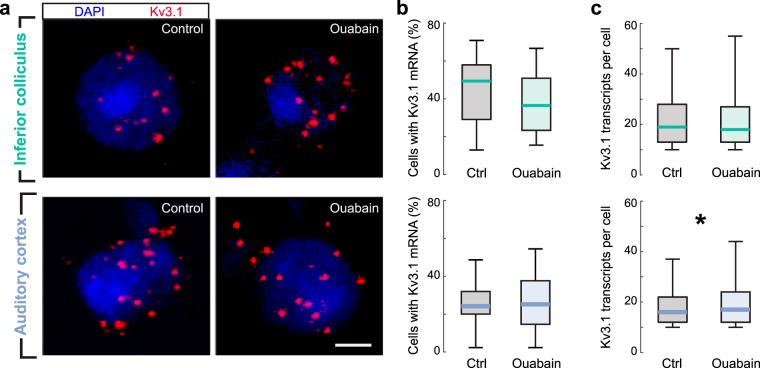
Kv3.1 mRNA is expressed in the inferior colliculus and auditory cortex, even after profound contralateral auditory nerve damage. Top row, inferior colliculus. Bottom row, auditory cortex. (**a**) Individual *KCNC1* mRNA transcripts that encode the Kv3.1 protein identified near DAPI-labeled nuclei of individual cells. Fluorescently labeled individual mRNA transcripts are identified within a fixed radius of each nucleus, counted, and then assigned to a given cell with automated software. Scale bars = 3 μm. (**b**) The percentage of DAPI + nuclei within a 185 μm^2^ region of interest that contain at least 10 mRNA transcripts are quantified in the ICc (top) or ACtx (bottom) 30 days after control or ouabain treatment (n = 36 imaging regions from 4 ouabain-treated mice and 27 imaging regions from 3 sham-treated mice). (**c**) Same as *B*, except the number of identified transcripts are quantified only in the Kv3.1+ cells (n = 2,420/2,814 in the ICc and 941/1,399 cells in ACtx for ctrl/ouabain, respectively). Plots in 5*B* and *5* *C* depict the median, interquartile range and 95% confidence intervals. Asterisk indicates p < 0.05 with a rank sum test; otherwise, pairwise differences between control (Ctrl) and ouabain are not significant.

Before assessing how AUT00063 affected auditory temporal processing in ouabain-treated mice, we first wanted to confirm that Kv3.1 was expressed in the mouse auditory midbrain and cortex^41,42^ and also to determine if expression levels were affected by partial denervation of the contralateral auditory nerve. To accomplish this, we quantified the percentage of Kv3.1+ cells in the ICc and ACtx as well as the expression levels within those cells using fluorescence *in situ* hybridization of mRNA for the Kv3.1 gene, *KCNC1* ^25,43,44^ (Fig. 5a). We positioned small regions of interest (185 μm × 185 μm) in coronal sections of the ICc and ACtx from mice that had either undergone contralateral auditory nerve damage with ouabain or a sham manipulation 30 days prior. We found that Kv3.1 mRNA was present in roughly 45% of ICc cells and 25% of ACtx cells, but the percentage of Kv3.1+ cells did not differ between control and ouabain-treated mice (rank sum test, p > 0.25 for both IC and ACtx, Fig. 5b top and bottom, respectively). Similarly, mRNA expression levels within identified Kv3.1+ cells were roughly equivalent following contralateral denervation (Fig. 5c). In fact, Kv3.1 mRNA levels in ACtx were slightly elevated above control mice (rank sum test, p = 0.02, ACtx; p = 0.09, ICc). These data suggested that AUT00063 may have available targets in the auditory midbrain and cortex, even after a profound loss of auditory nerve afferent fibers in the contralateral ear.

### Systemic delivery of AUT00063 improves auditory synchronization and decoding of stimulus modulation rate

**Figure 6:**
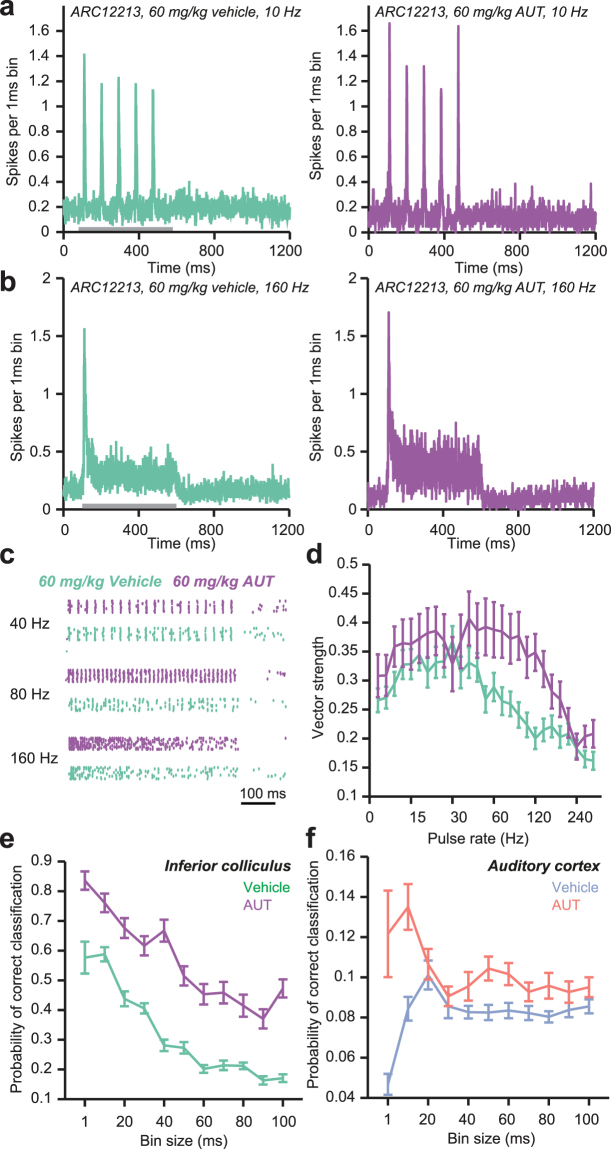
AUT00063, a novel Kv3.1 channel modulator, improves temporal coding in the IC as measured via responses to chirp train stimuli 30 days after ouabain. (**a**) Example PSTH from a multiunit recording in the ICc of a ouabain-treated mouse with vehicle (left, green trace) or AUT00063 (right, purple trace) injected systemically 20–25 minutes prior to recording. Stimulus is a 10 Hz chirp train presented at 20 dB above threshold. (**b**) Same as (**a**), but the stimulus is a 160 Hz chirp train. (**c**) Example raster plots of multiunit spiking responses across trials of chirp trains of various frequencies, with systemic delivery of vehicle (green rasters) or AUT00063 (purple rasters, n = 73 recording sites in both conditions in ICc). (**d**) Average vector strength of all significantly sound responsive sites across chirp train frequency, with and without AUT00063. (**e**) Probability of correct classification of chirp train frequency with increasing PSTH bin sizes for IC multiunit responses (significantly responding to sound), with and without AUT00063. (**f**) Same as (**e**), but for ACtx (n = 61 sites).

Findings from *in silico* and *in vitro* experiments suggested that AUT00063 could improve spike timing fidelity *in vivo*, perhaps mitigating temporal processing deficits that accompany auditory nerve damage. We tested this possibility by recording from a subset of ACtx and ICc multiunit recording sites described in Fig. 2 following a systemic injection of AUT00063 or vehicle (60 mg/kg, n = 61 in ACtx, n = 73 in ICc). In ouabain-treated mice injected with a vehicle solution, representative ICc units showed strong response adaptation and poor synchronization to trains of broadband acoustic pulse trains, particularly at high frequencies (Fig. 6a–c, left). Following AUT00063 injection, sound-evoked firing rates were more sustained and exhibited improved trial-by-trial synchronization, particularly at high pulse rates (Fig. 6a–c, right). Looking across the full range of pulse rates, we observed a significant increase in auditory synchronization accuracy across our sample of ICc recordings sites in mice injected with AUT00063, as compared to vehicle (F(1) = 20.2, *p* = 0.021, repeated-measures ANOVA, Fig. 6d). We then applied the PSTH-based classifier to recordings made in the vehicle and drug conditions to determine whether improvements in synchronization accuracy provided an improved basis for decoding stimulus pulse rate. Following the approach described in Fig. 2d–e, we computed the mean classification accuracy across the full range of pulse rates, when binning the PSTH at varying degrees of temporal resolution. We found that the improved synchronization accuracy in ICc was associated with improved classification accuracy both when precise spike timing information was available (small bin sizes) and when decoding was based on coarse changes in firing rate statistics over time (large bin sizes) (F(1) = 67.7, *p* < 0.01, repeated-measures ANOVA, Fig. 6e). In ACtx, where temporal coding is poor overall, AUT00063 significantly boosted decoding accuracy, particularly at fine temporal resolution where classification in the vehicle condition is at chance (F(1) = 7.75, *p* = 0.024, repeated-measures ANOVA, Fig. 6f).

## Discussion

Here we show that in a mouse model of auditory neuropathy, where more than 95% of Type-I auditory nerve fibers have degenerated following local application of ouabain (Fig. 1), temporal processing deficits in the midbrain persist even when auditory thresholds and level-dependent growth functions have partially recovered (Fig. 2). Recording from a principal input to ICc neurons in a brainstem slice preparation revealed that a newly developed Kv3.1 modulator, AUT00063, reduced variability in action potential timing in response to direct current injections (Fig. 3). Numerical simulations suggested that increasing the proportion of AUT-modified Kv3.1 conductance with AUT00063 would also improve synchronization to periodic synaptic inputs (Fig. 4). We observed robust transcription of Kv3.1 mRNA in the ICc and ACtx in normally hearing mice as well as their ouabain-treated counterparts, suggesting that AUT00063 could act on neurons at multiple stages of central auditory processing (Fig. 5). Returning to the intact preparation, we then asked whether systemic administration of AUT00063 could ameliorate the persistent temporal processing deficits in the midbrain of mice with cochlear neuropathy. We found that ICc synchronization to periodic auditory inputs was improved shortly after AUT00063 injections and that changes in both ICc and ACtx supported more accurate temporal decoding of stimulus modulation (Fig. 6). These findings suggest that temporal processing deficits following cochlear afferent neuron loss can be ameliorated with drugs that act only on central neurons, as Kv3.1 is not expressed in the auditory periphery.

Hearing loss engages a cascade of homeostatic adaptations in the central nervous system that sensitize central auditory neurons to a diminished bottom-up drive from the auditory periphery^13,14,45^. Given adequate time, this plasticity can fully restore sound-evoked cortical thresholds, frequency coding, sound level coding and even detection behaviors despite the absence of any measurable acoustic reflexes or brainstem evoked potentials^12,13,15,46,47^. However, there are profound perceptual consequences “hiding” behind normal detection thresholds such as tinnitus, reduced sound level tolerance, and poor discrimination of temporal fine structure, all of which are thought to arise from the loss of redundant afferent coding in the auditory periphery, the loss of high-fidelity temporal cue extraction in the auditory brainstem, or may arise directly from pathologically overpowered amplification in the central pathway^48–53^.

Whether describing loudspeaker systems, cochlear hair bundles, or recurrently connected central synapses, excess gain can bring oscillators closer to points of instability, leading to self-sustained “ringing” and distortion of the input signal timing^54–57^. A key goal in the context of hearing research is to coax central circuits back from unstable, hyperexcitable states by turning down central amplification. In theory, this could be achieved by restoring input from the periphery through some combination of repair or regeneration of cochlear hair cells and their synapses with cochlear neurons. Realistically, repairing an adult inner ear with chronic, cumulative, widespread forms of cellular degeneration due to a combination of aging, noise exposure and ototoxins is a very challenging problem that has yet to be demonstrated in humans^58^. An alternative would be to adjust the “volume knob” on central gain with drugs that decrease neuronal excitability in the central auditory pathway, such as benzodiazepines, but the efficacy of GABA agonists decrease over time and have many non-specific, unwanted targets^59–61^.

Our study was focused on a third option for reducing excess central auditory gain and improving temporal signal processing - targeting voltage-gated ion channels that regulate intrinsic membrane excitability. Whereas earlier approaches have focused on reducing central auditory gain through manipulations of KCNQ and HCN channels, which are active at subthreshold membrane potentials^62,63^, we focused instead on Kv3.1, a delayed rectifier channel open at depolarized membrane potentials, whose role is to shape the action potential^20,21^. Compared to most other delayed rectifier potassium channels, Kv3.1 channels activate more rapidly during the rising phase of the action potential and deactivate more rapidly at hyperpolarized membrane potentials^25^. Due to this rapid deactivation, neurons with Kv3.1 currents fire at high rates with narrow action potentials and lack the relative refractory period present in neurons with slowly deactivating outward potassium currents^64^. Fast-spiking neurons throughout the central auditory pathway express Kv3.1 and computational, genetic or pharmacological *reductions* of Kv3.1 conductances in central auditory neurons reduces spike timing precision and maximum sustained firing rates to periodic inputs^18,23^. Here, we applied a recently developed compound that *increased* Kv3.1 currents at less depolarized membrane potentials and confirmed that acute application improved action potential reliability and sound-evoked spike timing precision.

Whether and how these benefits persist in animal models of chronic AUT00063 dosing remains to be seen. In this regard, central nervous system plasticity is both a blessing and a curse. On the one hand, self-organizing systems can compensate for the loss of approximately 95% of peripheral inputs and still retain useful sensory coding and perceptual processing. On the other hand, the compensatory plasticity processes that pull individual neurons back to their homeostatic set points of excitability can lead to unstable, hyper-synchronized network states that distort temporal processing and even generate phantom percepts, as in the case of tinnitus. Chronic application of exogenous compounds that decrease excitability or improve stimulus coding in the short-term can themselves engage compensatory processes that would render potential therapies ineffective in the long-term. In this regard, approaches that reprogram neural set points of excitability through gene therapy rather than pharmacological approaches that continuously push against the tide of hyperexcitability may also prove useful.

## Materials and Methods

### Adult cochlear denervation

All procedures were approved by the Animal Care and Use Committee of the Massachusetts Eye and Ear Infirmary and in accordance with guidelines established by the NIH for the care and use of laboratory animals. CBA/CaJ mice (n = 15) were anesthetized with ketamine (120 mg/kg) and xylazine (12 mg/kg), with half the initial ketamine dose given when required. The connective tissue, underlying muscle and the facial nerve were blunt dissected away from the bulla with retractors. A small opening was made in the bulla with the tip of a 28.5-gauge needle. The exposed round window niche was filled with 1–2 µL of the ouabain solution using a blunted needle. Ouabain was reapplied five more times at 15-minute intervals, wicking the existing solution away before each application. Preliminary measurements of the ABR and DPOAE were made after the sixth application to confirm an immediate ABR threshold shift without any change in DPOAE thresholds or amplitudes (see below for cochlear function testing procedures). Additional rounds of ouabain solution were applied, as necessary, until the ABR threshold at 16 kHz was 55–70 dB SPL (for ouabain plus sterile water) or remained at control levels (25–35 dB SPL, for sterile water alone). The incision was sutured and the mouse was given buprenorphine as an analgesic before transfer to a warmed recovery cage (0.5 mg/kg).

### Cochlear function testing

ABR measurements were performed with transdermal electrodes under ketamine/xylazine anesthesia approximately 30 days after ouabain treatment. Core body temperature was maintained at 36.5**°** C with a homeothermic heating pad. ABR stimuli were tone pips, 5 ms in duration (8, 16, and 32 kHz, from 20–80 dB SPL in 5 dB steps, 0.5 ms raised cosine onset and offset ramps). ABR wave 1b was identified manually and amplitude calculations were performed with custom software (LabView). ABR threshold was defined as the lowest stimulus level at which a repeatable waveform morphology could be visually identified. Visual identification of the waveform was validated with a semi-automated algorithm that identifies peaks and troughs of putative ABR waves by first calculating the negative zero crossings (NZCs) of the first derivative of the recorded waveform. To avoid mislabeling peaks in noisy signals, the algorithm eliminates spurious peaks by setting a threshold for NZC amplitude based on the noise floor, calculated from the standard deviation of the first 1 ms of the signal^65^. The 2f1-f2 DPOAE was measured in the ear canal using primary tones with a frequency ratio of 1.2 and level difference of 10 dB, incremented in 5 dB steps from 20–80 dB SPL. All ABR and DPOAE data has been published in an earlier report^12^.

### Drug delivery and testing

AUT00063 ((5 R)-5-ethyl-5-methyl-3-[2-({4-methyl-3-[(trifluoromethyl)oxy]phenyl}oxy)-5-pyrimidinyl]-2,4imidazolidinedione) was provided by Autifony Therapeutics, Ltd (UK). It was prepared in the following vehicle: Captisol 20% w/v, HPMC 0.5% w/v, Tween80 0.1% w/v in water. In order to achieve a homogeneous suspension for i.p. dosing, the drug and vehicle suspension was vortexed twice and briefly sonicated before being drawn into the syringe and immediately injected into the animal. The animal was given 15–20 minutes to recover after i.p. injection before recordings began, in accordance with maximal drug absorption (Autifony Therapeutics, personal communication). Vehicle and drug conditions were tested in random order on separate days in individual mice.

### Unit recordings in IC and ACtx through chronically implanted electrodes

Procedures for chronic extracellular recordings for these animals have been described previously^12^. Mice (n = 4 ouabain-treated and n = 4 sham-treated) were maintained at 36.5 °C and brought to a surgical plane of anesthesia with ketamine/xylazine as described above. Using a scalpel, a craniotomy (1–4mm^2^) was performed over the right primary auditory cortex and right central nucleus of the inferior colliculus, leaving the dura mater intact. The brain surface was covered with clear sterile ointment. Chronic implants consisted of multichannel silicon probes (177 µm^2^ contact area, 100 µm contact separation; NeuroNexus Technologies) arranged in a 4 × 4 configuration mounted onto a bidirectional microdrive (for ACtx) or 16 × 1 stationary probe configuration (all IC, some ACtx). The ACtx implant was positioned by first mapping the cortex to delineate the low-high-low caudal-rostral best frequency (BF) gradient that uniquely identified the orientation of the primary auditory cortex and the anterior auditory field^66,67^. The ICc was identified based on a low-to-high best frequency gradient along the dorsal-ventral axis of a single shank multichannel probe. Bone wax (3 M) was packed around the margins of the craniotomy to protect the probes and brain surface, and the microdrive (for ACtx implants) and headstage connector were affixed to the skull surface using acrylic bonding material (C&B MetaBond, Parkell). Ground wires were implanted outside of the auditory cortex and the mouse was given subcutaneous post-surgical injections of buprenorphine (0.05 mg/kg) and sterile saline (0.5 cc) to reduce pain and dehydration, respectively. A clear plastic cylinder was affixed to the perimeter of the ACtx craniotomy to protect the probes and brain surface.

### Stimuli

Stimuli were generated with a 24-bit digital-to-analog converter (National Instruments model PXI-4461). For DPOAE and ABR tests, as well as during surgeries, stimuli were presented via in-ear acoustic assemblies consisting of two miniature dynamic earphones (CUI CDMG15008–03 A) and an electret condenser microphone (Knowles FG-23339-PO7) coupled to a probe tube. Stimuli were calibrated at the tympanic membrane in each mouse before recording. For awake recordings, stimuli were presented via a free-field electrostatic speaker (Tucker-Davis Technologies) placed approximately 10 cm from the left ear canal. Free-field stimuli were calibrated before recording using a wide-band ultrasonic acoustic sensor (Knowles Acoustics, model SPM0204UD5).

### *In vivo* neurophysiology data collection

Raw signals were digitized at 32-bit, 24.4 kHz (RZ5 BioAmp Processor; Tucker-Davis Technologies) and stored in binary format. Subsequent analyses were performed in MATLAB (MathWorks). The signals were notch filtered at 60 Hz and then bandpass filtered at 300–5000 Hz with a fifth-order acausal Butterworth filter. Multiunit spiking activity was limited to spike waveforms that were at least 3.5 standard deviations above the mean of a 10 s running average for each electrode (OpenEx, Tucker-Davis Technologies). We employed an online automated algorithm to remove the common mode signal from the multiunit spike record. The common mode signal was calculated by averaging across all recording channels, and the rejection was achieved by subtracting the averaged signal from each individual channel^68^. Acoustic stimulation was restricted to the left (treated) ear. While mice were briefly anesthetized with isoflurane, a dense foam earplug (3M), cut to fit the ear canal of a mouse (~2 mm diameter) was securely fit into the external auditory meatus of the right ear. We have previously confirmed that this unilateral earplug provides at least 60 dB of attenuation across the frequencies tested^12^. All baseline measurements (i.e., before drug delivery) have been published in an earlier report^12^.

### Animals and slice preparation

CD-1 mice of either sex (postnatal day 15–19, n = 8), purchased from Charles River Italy, were deeply anaesthetized and decapitated. Coronal brainstem slices (200 μm) containing the dorsal cochlear nucleus (DCN) were obtained CD-1 mice in low Na+ artificial cerebrospinal fluid (ACSF) containing in mM: KCl (2.5), CaCl_2_ (0.1), NaH_2_PO_4_ (1.2), MgCl_2_ (4), NaHCO_3_ (26), sucrose (25) and glucose (10), ascorbic acid (0.5) gassed with 95% O_2_-5% CO_2_. Slices were then incubated in normal ACSF at 37 °C in a slice chamber. After 1 hour, the chamber was removed from the water bath, compound (AUT00063 30 µM or DMSO (0.1%) was added to the slice chamber (1 h incubation). Recordings were carried out at room temperature in normal ACSF containing in mM: NaCl (125), KCl (2.5), CaCl_2_ (4), NaH_2_PO_4_ (1.2), MgCl_2_ (2), NaHCO_3_ (26), Na- pyruvate (2), Myo-inositol (3), Ascorbic acid (0.5) and glucose (10), and saturated with 95% O_2_-5% CO_2_.

### *In vitro* electrophysiological recordings

All procedures were approved by the Ministero della Salute (Italy, art.12 del D.lgs. 116/92). Patch-clamp recordings were carried out in current clamp mode in a submersion chamber mounted on an upright microscope (Axioskop2FS, Carl Zeiss, Germany). Neurons were visualized with a 40X objective using infrared-differential interference contrast (IR-DIC) video microscopy (Hamamatsu C4742-96, Hamamatsu City, Japan). Fusiform principal neurons were identified on the basis of the distinctive somatic morphology. Whole-cell recordings were performed using pulled borosilicate-glass patch pipettes filled with an internal solution containing (in mM): KCl (110), EGTA (0.2), HEPES (40) MgCl_2_ (1), Na-phosphocreatine (5) CaCl_2_ (0.1); pH 7.3 adjusted with KOH to reach tip resistance of 3–7 MΩ. Recordings were filtered at 3 kHz and sampled at 50 kHz (Multiclamp 700B amplifier, Molecular Devices LLC, Sunnyvale, CA, USA). Stimulation was controlled through a Digidata 1440 A interface (Molecular Devices LLC, Sunnyvale, CA, USA) using pClamp 10.3 software. Capacitive transients were neutralised and series-resistance was monitored continuously throughout the experiment. Neurons were excluded from further analysis if the series resistance changed by more than 20% over the course of recording.

Data were analysed by using Clampfit 10.3 software (Molecular Devices LLC, Sunnyvale, CA, USA). Action potential timing was measured with the single step protocol as the latency between the start of the trace and the peak. The coefficient of variation was defined as the ratio of the standard deviation σ to the mean μ. The afterhyperpolarization amplitude was measured as the peak negativity following the action potential peak. The half width and rise time (10–90%) of the action potentials were calculated with clampfit using the event detention tool. The action potential amplitude was measured relative to the holding membrane potential (−70mV).

### Tissue Acquisition and *in situ* Hybridization

A quantitative analysis of mRNA level of the gene that encodes the Kv3.1 protein (*KCNC1*) was performed on seven additional mice (4 ouabain-treated and 3 sham-treated) that showed equivalent hearing loss (or lack thereof) based on cochlear function testing. Approximately 30 days after manipulation of the contralateral ear (either sham or ouabain treatment), brains were extracted and flash frozen in liquid nitrogen, and then embedded in OCT (TissueTek, VWR, Radnor, PA). Embedded brains were secured in a −28° cryostat and cut into 10 μm coronal sections. Frozen sections were mounted on pre-chilled (−20C) Superfrost slides (Fisher Scientific, Waltham MA) and stored at −80°. Fluorescence *in situ* hybridization for *KCNC1* mRNA (transcript variant B) was performed on sections containing the auditory cortex or the inferior colliculus. Custom target probes were provided by Advanced Cell Diagnostics (ACD, accession no. NM_008421.3, Hayward CA, USA), as previously described^69^. Tissue permeabilization, mRNA hybridization and amplification, and fluorescent labeling were all performed using the RNAscope Multiplex Fluorescent Reagent Kit and HyBEZ oven according to the manufacturer’s instructions for fresh-frozen brain tissue (ACD, Hayward CA, USA). Cell nuclei were counterstained with DAPI and sections were coverslipped with Vectashield (Vector Labs, Burlingame CA).

### Data analysis

Sound-driven sites were identified by binning the PSTH at 10 ms resolution, and determining if at least one bin within the stimulus presentation window (starting at 0 ms re: presentation and ending 50 ms after stimulus offset) was at least 3 SDs above the spontaneous firing rate distribution.

Rate-level functions were calculated from responses to broadband chirp stimuli. Responses were smoothed with a 3-point moving average and fit with a six parameter Gaussian function^70^.

Frequency-modulated chirps were 1 ms in duration, and spanned 4–64 kHz. The FM rate was calculated to compensate for the mouse basilar membrane group delay in order to generate a synchronous, equipotent displacement of the cochlear partition^35^. Vector strength of responses to chirp trains was calculated as follows^36^:

1 $${\rm{VS}}=\frac{\sqrt{{(\sum _{i=1}^{n}\cos \theta )}^{2}+{(\sum _{i=1}^{n}\sin \theta )}^{2}}}{n}$$

where VS is the vector strength, n is the number of spikes over all trials, and θ is the phase of each spike in radians. Phase-projected vector strength is calculated as follows:

2 $${\rm{VSpp}}={{\rm{VS}}}_{{\rm{t}}}\,\cos ({\phi }_{{\rm{t}}}-{\phi }_{c})$$

where VSpp is the phase-projected vector strength per trial, VS_t_ is VS per trial, and ${\phi }_{t}$ and ${\phi }_{c}$ are the trial-by trial and mean phase angle in radians. Cycle-by-cycle vector strength, a metric that describes both the degree of synchronization and the reliability of the synchronization across the duration of the stimulus, is calculated similarly to VSpp, except that it is computed for each cycle individually rather than over the entire stimulus period. A VSpp value is generated per cycle, and VSpp values over all cycles are averaged together to generate the VScc.

#### PSTH classifier model

The PSTH classifier model compares the Euclidean distance between the population single trial spike train elicited by a given stimulus to the response templates created for each stimulus^37^. The spike train is classified as being generated in response to the stimulus from which its distance is minimal. A response window of 0.1 s was aligned with stimulus onset. A matrix with *T* × *S* rows and *B* × *N* columns was constructed, where *T* is the number of stimulus repeats (n = 20), *S* is the number of stimuli, *B* is the number of bins that contain spikes, and *N* is the number of recording sites in the ensemble (1–20 units).

Let v_i,j_ represent the spike count in *i*th row and *j*th column of the matrix, where *i* goes from 1 to *ST* and *j* goes from 1 to *NB*. Templates for each stimulus were defined as ${\bar{v}}^{s}=[{\bar{v}}_{1}^{s},\ldots ,{\bar{v}}_{NB}^{s}]$, where the jth element is calculated as

3 $${\bar{v}}_{y}^{j}=\frac{1}{T}\,\sum _{i\in s}{v}_{{\rm{ij}}}^{s}$$

for each trial ${v}^{i}=[v{}_{i,1},\ldots ,{v}_{i,B}]$, the Euclidean distance between that trial and each stimulus template ${\bar{v}}^{s}=[{\bar{v}}_{1}^{s},\ldots ,{\bar{v}}_{B}^{s}]$ was defined as

4 $${d}_{s}^{i}=\sqrt{\sum _{j=1}^{NB}{({v}_{i}{,}_{j}-{\bar{v}}_{j}^{s})}^{2}}$$

Using these distances, the spike train is classified as being generated by the stimulus class represented by the closest template, resulting in an outcome vector of $c=[{c}_{1},\ldots ,{c}_{ST}]$, where

5 $${c}_{i}=\mathop{\text{arg}\,\min }\limits_{s}({d}_{s}^{i})$$

Therefore, *c* will be of the same length as *ST* (total number of trials for all stimuli), and each element *c*_*i*_ indicates to which stimulus the *i*th trial is assigned.

### Computational Modelling

A simplified model neuron was used. Responses were simulated by integration of the equation:

6 $$C\frac{dV}{dt}=Isyn(t)-{I}_{Na}-{I}_{Kv}$$

where *I*_*Na*_ represents Na^+^ current, *I*_*Kv*_ represents Kv3.1 current and *Isyn(t)* is the synaptic current. The capacitance C was 0.2 nF. Equations for *I*_*Na*_ and *I*_*Kv*_ were similar to to those in Macica *et al*.^23^. Specifically, *I*_*Na*_ = *g*_*Na*_ *m* ^3^ *h* (V − 50) and *I*_*Kv*_ = *g*_*Kv*_ *n* ^3^p (V + 80). The evolution of the variables m, h, n, and p were given by equations of the form

7 $$\frac{dj}{dt}={\alpha }_{j}(1-j)-{\beta }_{j},$$

where,

8 $${\beta }_{j}={k}_{\beta j}\exp ({\eta }_{\beta j}V)$$

and j = m, h, n, p.

Kinetic parameters for the evolution of the variables m and h were g_Na_ = 4·0 µS, k_αm_ = 76.4 ms^−1^, η_αm_ = 0.037 mV^−1^, k_βm_ = 6.93 ms^−1^, η_βm_ = −0.043 mV^−1^, and k_αh_ = 0.000135 ms^−1^, η_αh_ = −0.1216 mV^−1^, k_βh_ = 2.0 ms^−1^ and η_βh_ = 0.0384 mV^−1^. For the unmodified Kv (Kv3.1) current, *g*_*K*v_ = 3·01 µS, with k_αn_ = 0.2719 msec^−1^, η_αn_ = 0.04 mV^−1^, k_βn_ = 0.1974 msec^−1^ η_βn_ = 0 mV^−1^, k_αp_ = 0.00713 ms^−1^, η_αp_ = −0.1942 mV^−1^, k_βp_ = 0.0935 ms^−1^ and η_βp_ = 0.0058 mV^−1^. For Kv3.1 with altered voltage-dependence and kinetics (modified low-threshold), the parameters for K_v_ were altered to those used previously to mimic the effects of AUT compounds (Brown *et al*., 2016) by changing the values of k_αn_ and k_βn_ to 0.25 msec^−1^ and 0.09197 msec^−1^, respectively. Synaptic currents were given by

9 $${I}_{syn}={g}_{syn}(V)\exp ({\rm{t}}/{\rm{\tau }})$$

where *g*_*syn*_ = 0.02 µS and τ = 40 ms, and were applied at 20 Hz.

### Kv3.1 mRNA image acquisition and analysis

Three regions of interest (185 μm × 185 μm each) were imaged at three different caudal-rostral positions within the IC and ACtx. In the ICc, all 3 images from each section were located within the central nucleus of the IC (ICc). In the ACtx, the 3 images from each section represented the superficial (centered on layer 2/3), middle (centered on layer 4), and deep (centered on layer 5/6) depths. Images were acquired with a Leica SP8 confocal microscope with a 63 × 0.8NA immersion lens and two Leica HyD detectors. Image stacks were transferred to image-processing software (Amira, Visage Imaging) and projected in three dimensions. Perimeters of DAPI-labeled nuclei were identified by vectors of maximal intensity contrast between the DAPI-labeled region and image background, which consistently demarcated the outer edge of the labeled nucleus. Neighboring DAPI nuclei that were not segregated based on intensity contrast were manually separated using the Volume Edit tool in Amira. Large clusters of overlapping nuclei that could not be manually separated were excluded from analysis. Partial segments of DAPI nuclei, located along the image border or above and below the imaging planes, were also excluded from analysis. In the *KCNC1* fluorophore channel, clusters of fluorescent pixels with a minimum diameter of 0.7–0.9 μm (4–5 pixels) were classified as individual *KCNC1* puncta. Single fluorescent clusters correspond to single mRNA copies, as previously described^71^. Large pixel clusters that could be visually separated into individual puncta were also subdivided using the Volume Edit tool in Amira; clusters that exceeded 1500 nm (8–10 pixels) and could not be visually separated were excluded from analysis. Identified *KCNC1* puncta within 2.5–3.5 μm (15 to 20 pixels) of a DAPI-labeled perimeter were then counted and assigned to that cell using the connected components function in Amira. Transcript counts per cell were then exported to MATLAB (Mathworks). We operationally defined the percentage of *KCNC1*+ cells by first identifying all DAPI+ nuclei fully contained within the imaging area and then expressing the fraction of nuclei that contained at least 10 *KCNC1*+ puncta as a percentage. *In situ* mRNA counts were not normally distributed and therefore statistics were performed using the rank sum test, a non-parametric comparison for unpaired samples.

### Data Availability

The datasets generated during and/or analysed during the current study are available from the corresponding author on reasonable request.
